# Supplementary material for: Chromothripsis during telomere crisis is independent of NHEJ, and consistent with a replicative origin
Source: Genome Res. 2019 May;29(5):737–49. doi: 10.1101/gr.240705.118 (PMC6499312; doi:10.1101/gr.240705.118)
Supplement: Supplemental Material [file supp_gr.240705.118_Supplemental_file_1.zip › contigs/annotated_contigs/DB48/contig.2.DB48_length_548_mean_cov_4.65875912409.docx]

**DB48_length_548_mean_cov_4.65875912409**

```
CACTTCCAACAACAGCAAATACATATTTTTTAAGTGCACATGAAATGATCACCAGAATAGAATTTATTCTTGGCCAAAAAACAACCTTT
 >chr7:49784991-49785319 + E=8e-186
AACAAATACAAAATATTAAAATTATACTAACAATGTTCTTGGACCATAGTAAGAAAAAAGTAGAAATGAACAGAAAAATATATACATAG

GAATATCCAAATATTTGAAAAATACTTTTACATAACCCATAGCACAAAGAAGCAGTTACACAAGATAATAGGAAATACCTTAAGTTTAA

TGAAAACAAAATCACAACGCATTGAAATTTGTGGGATACAAAACCAGTGCTTAAAGAA|AAA|GCAGTCTTGAGTGTCTGGATTCAACA
                                                           >chr7:49856336-49856559 - E=
GTGGGCTCTTGACTTCCTTAATCCTCATATTAAGCCCACAGGGAATGAATGGAAGATTGTCCCAATTTTGTAAGTGAAGAAACTGAAGA
1e-122
GGGGAAGCAAATTAGACAAGGTGATAGGGATAAAGCTAGAATGTGCCAGTCTAGCTCCAAAGCCAAGGCTTTTTGTACTATTTCTTCAA

GGAATTCAGTCTAACT
```
